# Supplementary material for: Neighbor-specific gene expression revealed from physically interacting cells during mouse embryonic development
Source: Proc Natl Acad Sci U S A. 2023 Jan 3;120(2):e2205371120. doi: 10.1073/pnas.2205371120 (PMC9926237; doi:10.1073/pnas.2205371120)
Supplement: Supplementary file 1 — Appendix 01 (PDF) [file pnas.2205371120.sapp.pdf]

Neighbor-specific gene expression revealed from physically interacting cells during mouse embryonic development

Supplementary Materials

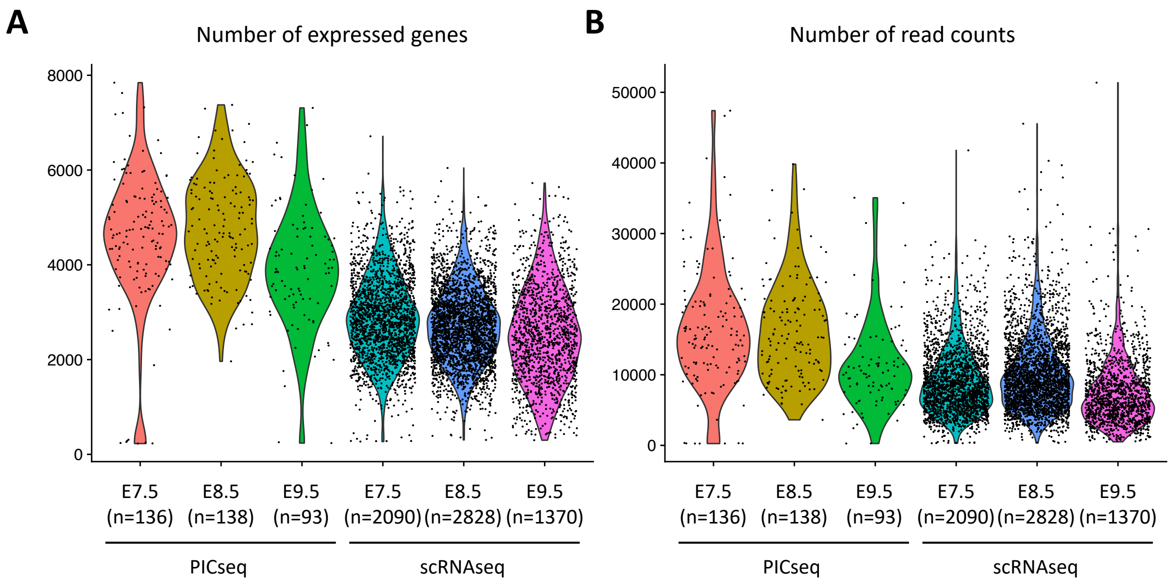

Figure 1. The quality control of PIC-seq and scRNAseq data of the mouse embryo at E7.5-E9.5. (**A**) The number of expressed genes and (**B**) the number of read counts in each PIC and cell for each sample.

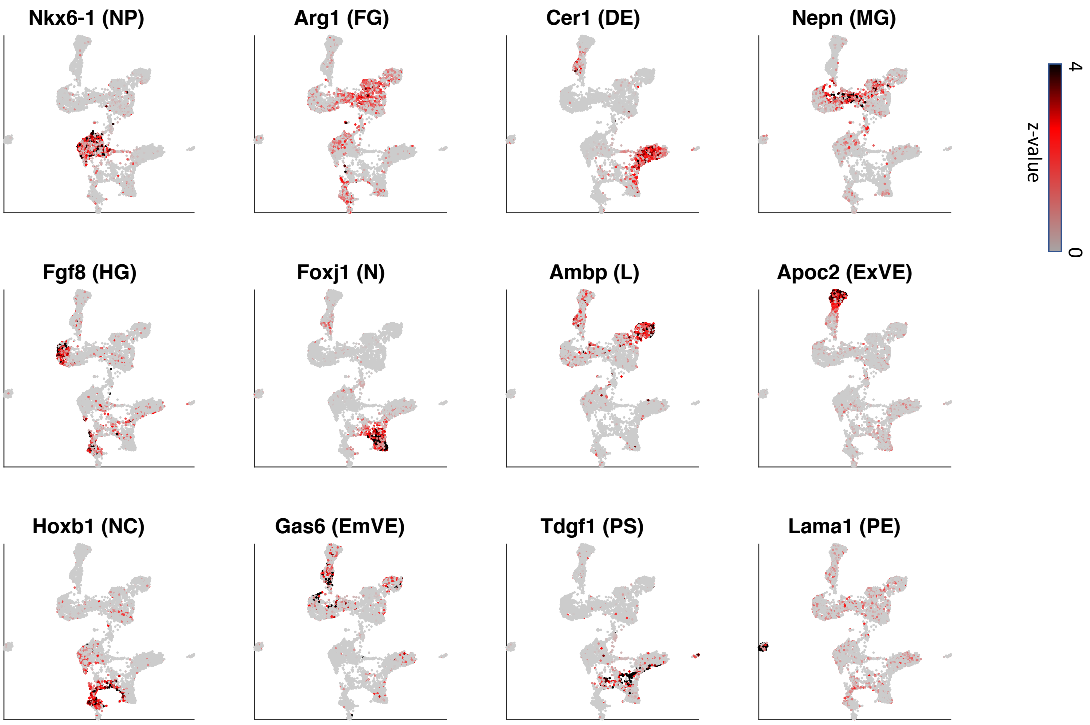

Figure S2. Expression profiles of 12 cell-type marker genes on a two-dimensional plot. NP: Neural progenitors, FG: Foregut, DE: Definitive endoderm, MG: Midgut, HG: Hindgut, N: Node, L: Liver, ExVE: Extra-embryonic visceral endoderm NC: Notochord, EmVE: Embryonic visceral endoderm, PS: Primitive streak, PE: Parietal endoderm. The expression levels of a marker gene are shown for the corresponding cell type.

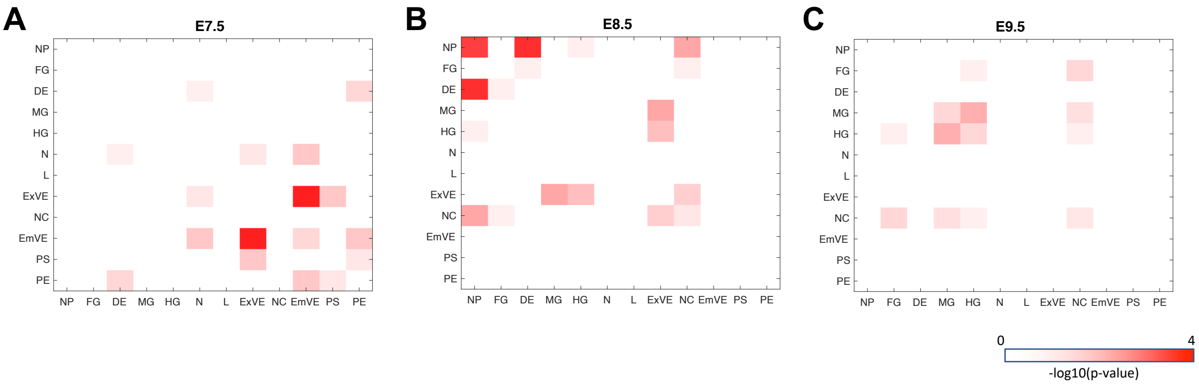

Figure S3. The significance of the frequencies of co-occurring cell types in PIC-seq compared with erroneous doublets found in scRNAseq at (**A**) E7.5, (**B**) E8.5 and (**C**) E9.5. The identified interactions from PIC (e.g. ExVE+EmVE at E7.5 and NP+DE at E8.5) were significantly more frequent than the erroneous doublets.

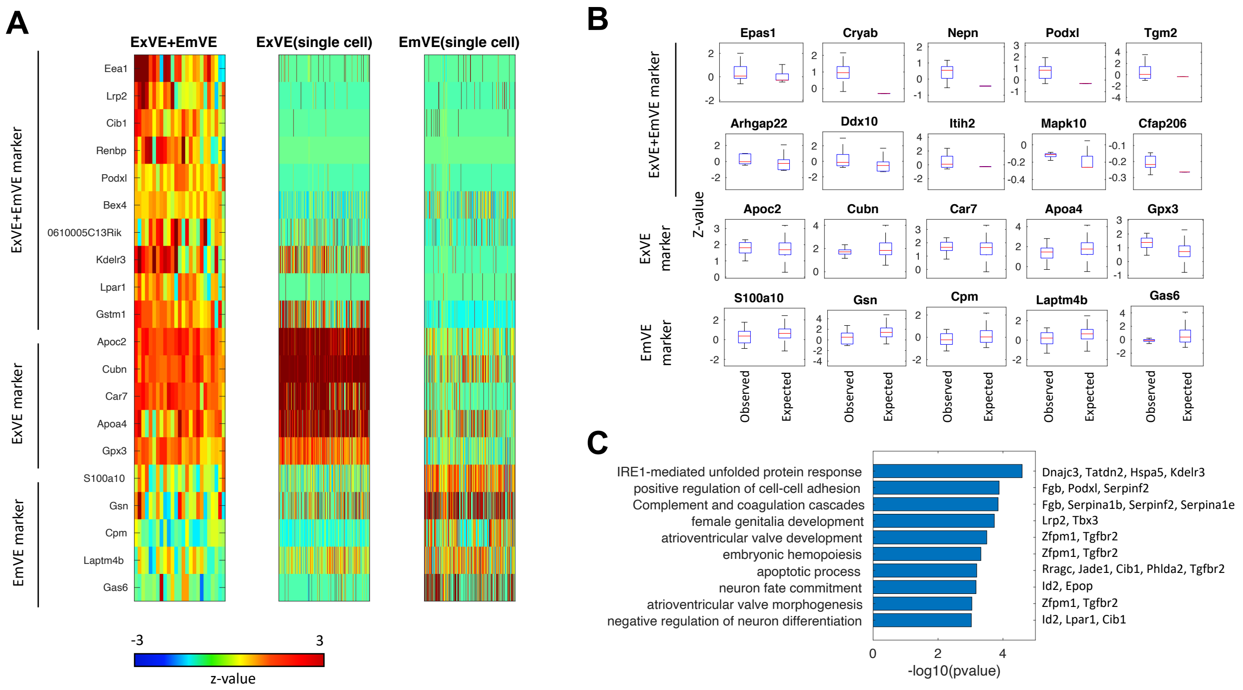

Figure S4. PIC-seq analysis identified genes highly expressed in ExVE+EmVE. (**A**) A heatmap of PIC-seq for ExVE+EmVE showed contact specific expression as well as the marker genes for each cell type. (**B**) The expression levels for the contact specific genes are significantly higher in the PIC-seq compared with expected expression levels obtained from scRNAseq. (**C**) GO and KEGG pathway terms enriched in the contact-specific genes for ExVE+EmVE.

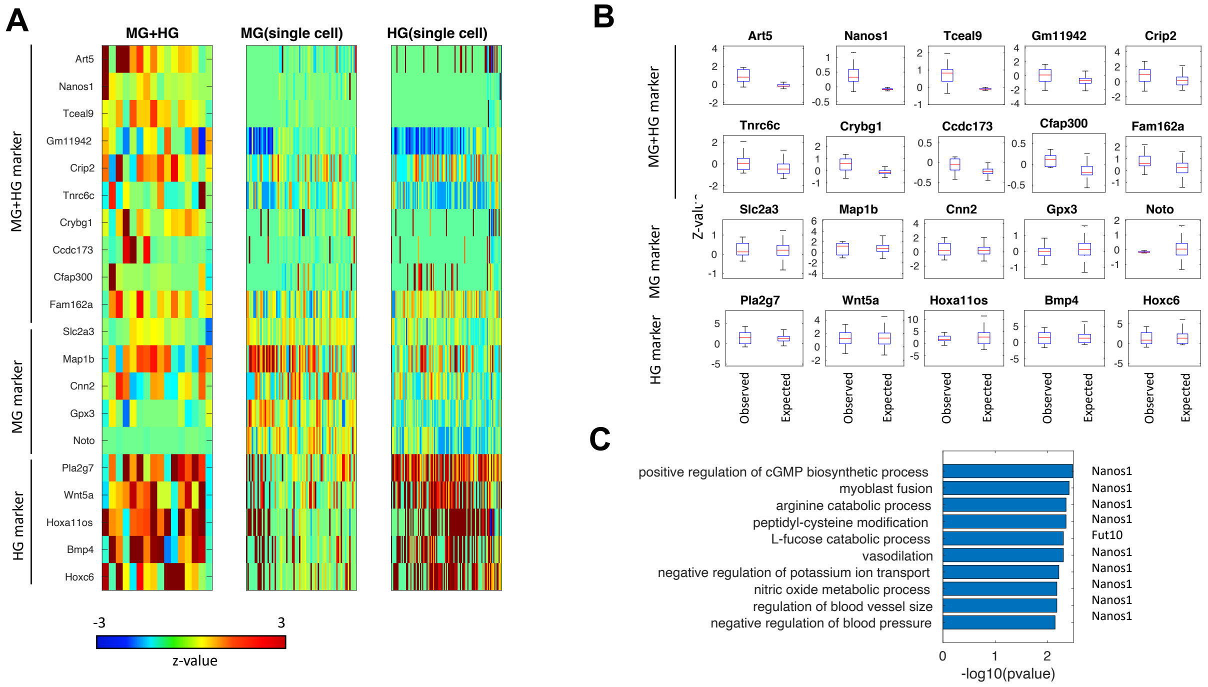

Figure S5. PIC-seq analysis identified genes highly expressed in MG+HG. (**A**) A heatmap of PIC-seq for MG+HG showed contact specific expression as well as the marker genes for each cell type. (**B**) The expression levels for the contact specific genes are significantly higher in the PIC-seq compared with expected expression levels obtained from scRNAseq. (**C**) GO and KEGG pathway terms enriched in the contact-specific genes for MG+HG.

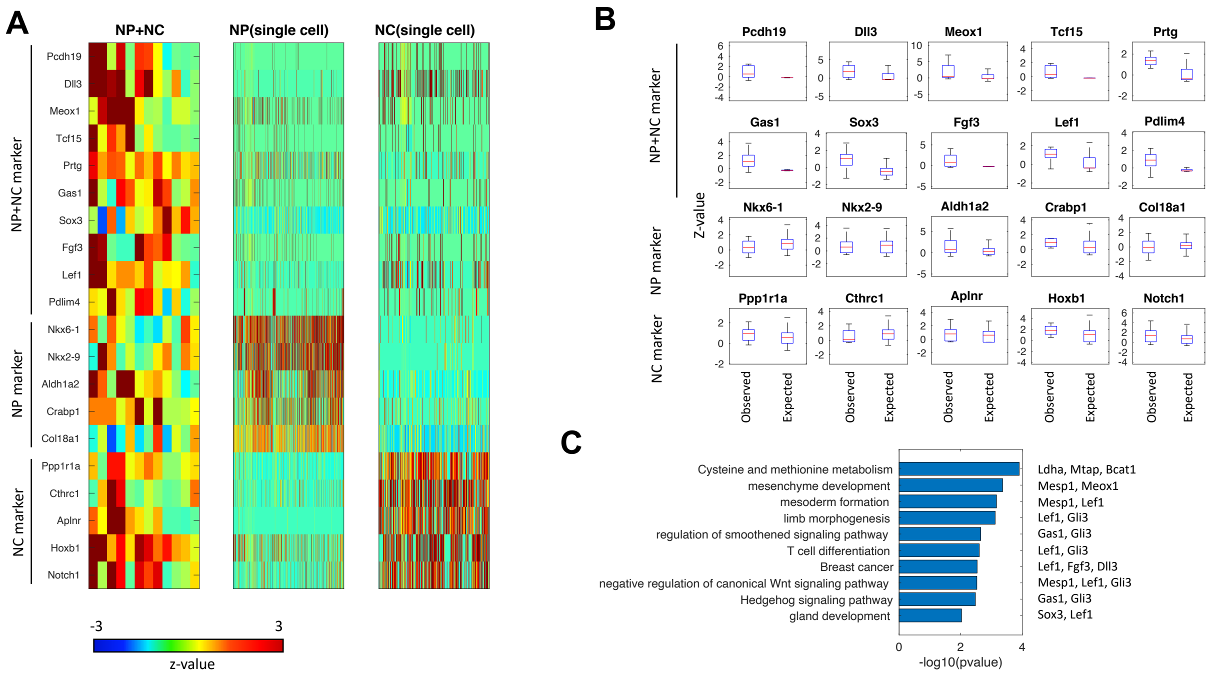

Figure S6. PIC-seq analysis identified genes highly expressed in NP+NC. (**A**) A heatmap of PIC-seq for NP+NC showed contact specific expression as well as the marker genes for each cell type. (**B**) The expression levels for the contact specific genes are significantly higher in the PIC-seq compared with expected expression levels obtained from scRNAseq. (**C**) GO and KEGG pathway terms enriched in the contact-specific genes for NP+NC.

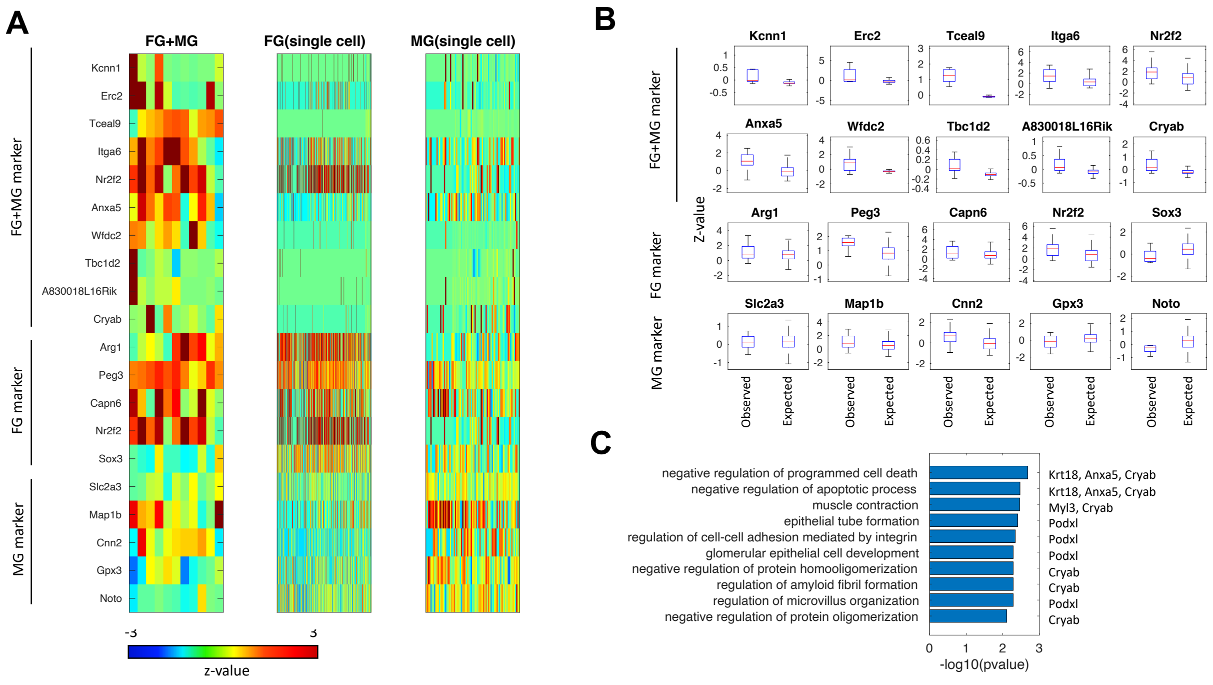

Figure S7. PIC-seq analysis identified genes highly expressed in FG+MG. (**A**) A heatmap of PIC-seq for FG+MG showed contact specific expression as well as the marker genes for each cell type. (**B**) The expression levels for the contact specific genes are significantly higher in the PIC-seq compared with expected expression levels obtained from scRNAseq. (**C**) GO and KEGG pathway terms enriched in the contact-specific genes for FG+MG.

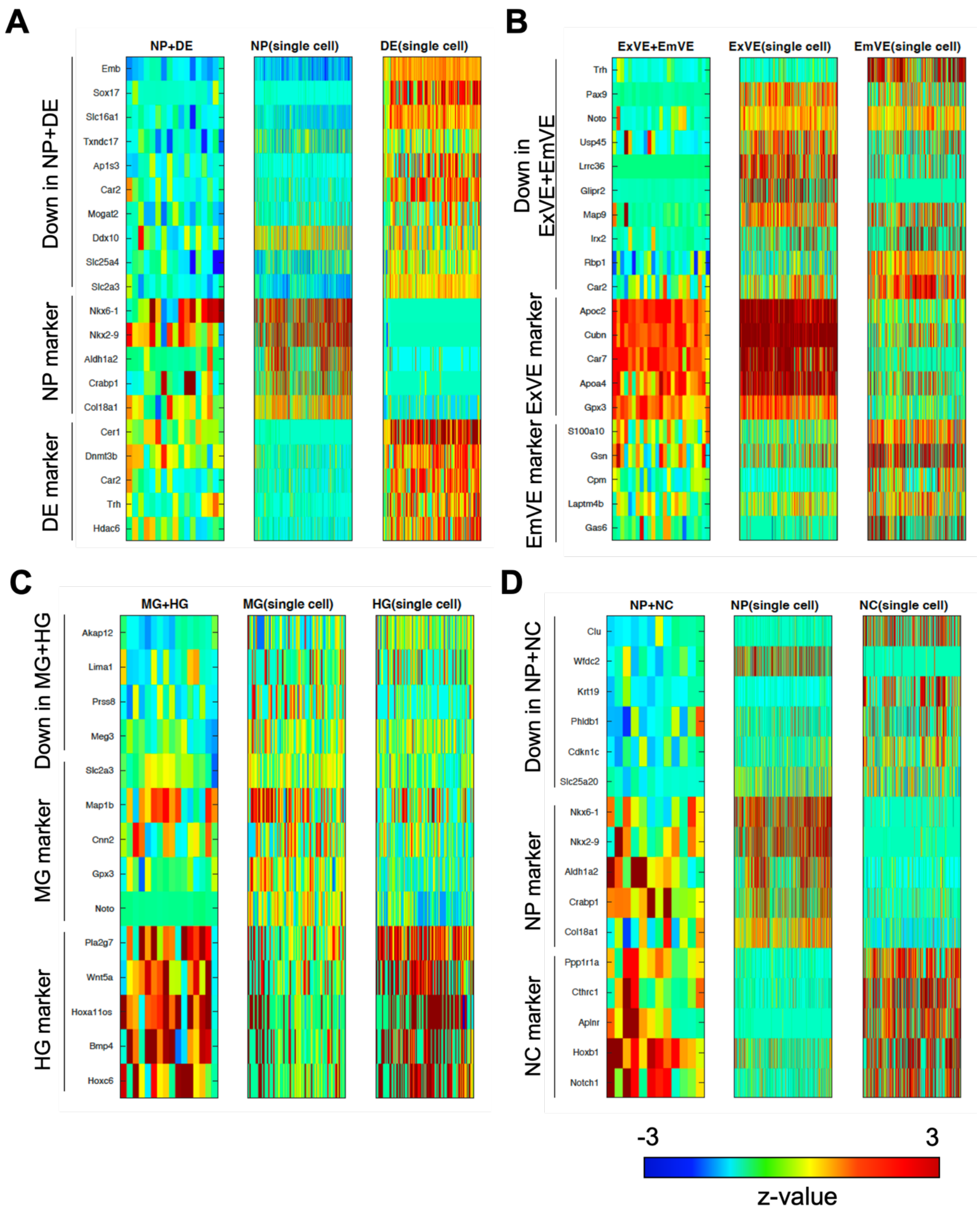

Figure S8. PIC-seq analysis identified genes lowly expressed in PICs compared to scRNAseq for individual cell types. (**A**) downregulated genes in the NP+DE PICs (**B**) downregulated genes in the ExVE+EmVE PICs. (**C**) downregulated genes in the HG+MG PICs. (**C**) downregulated genes in the NC+NP PICs.

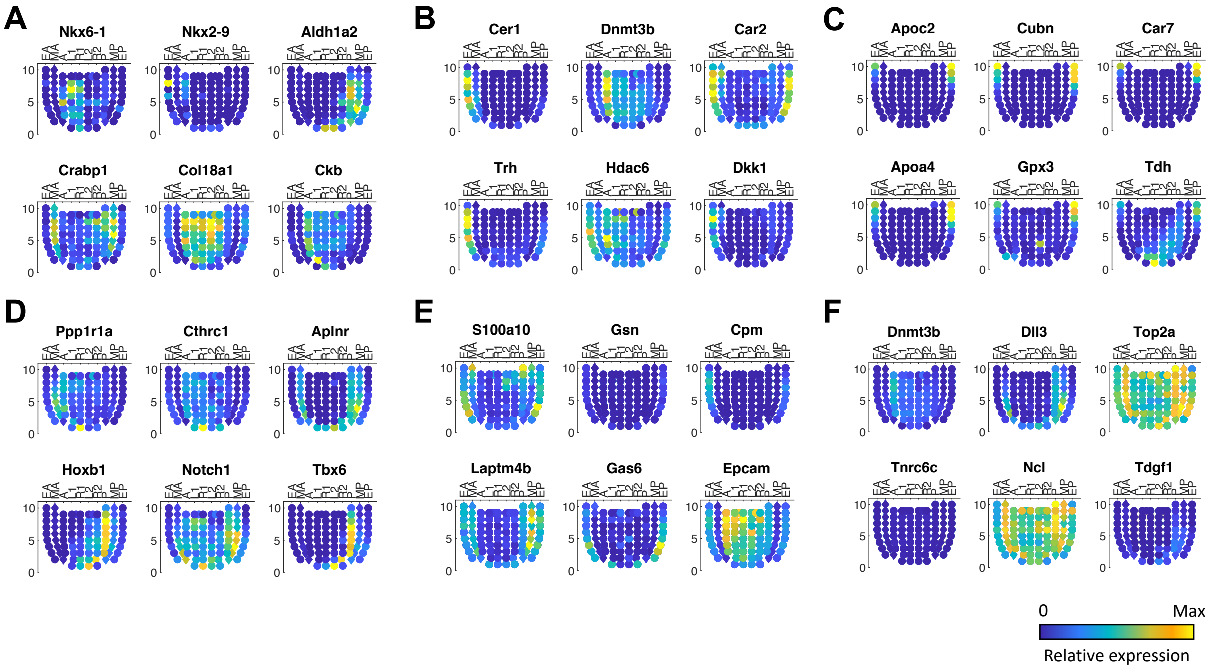

Figure S9. Expression levels of top 6 marker genes on Geo-seq data for each cell type (**A**) NP, (**B**) DE, (**C**) ExVE, (**D**) NC, (**E**) EmVE, (**F**) PS. While some of these markers are not expressed in the correct tissue, we assume that this is because the marker sets span E7.5-9.5, whereas these corn plots are based on data from E7.5 embryos. EA: Anterior Endoderm; MA: Anterior Mesoderm; A: Anterior; L1: Anterior Left Lateral; R1: Anterior Right Lateral; L2: Posterior Left Lateral; R2: Posterior Right Lateral; P: Posterior; MP: Posterior Mesoderm, EP: Posterior Endoderm.

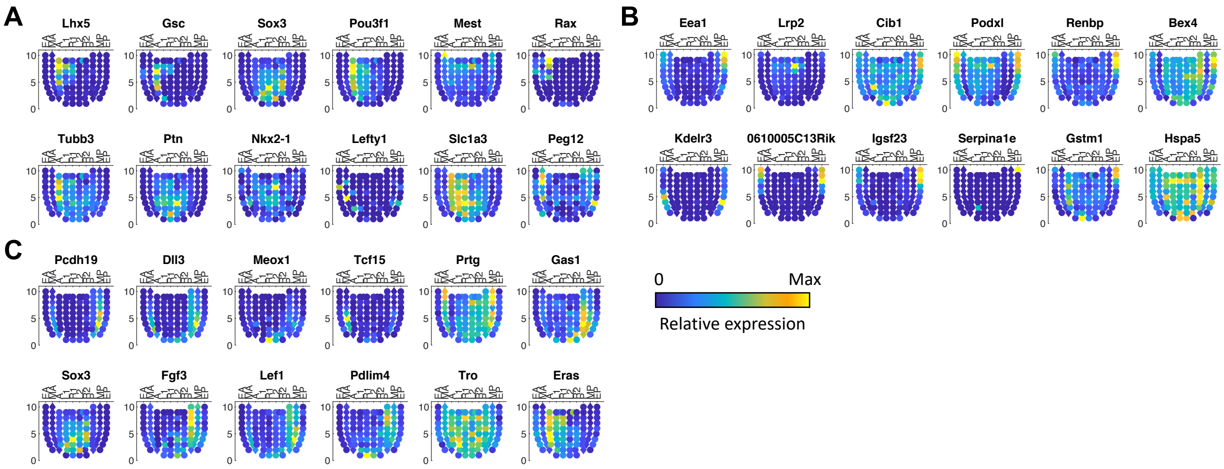

Figure S10. Expression levels of top 12 contact specific marker genes on Geo-seq data. (**A**) NP+DE, (**B**) ExVE+EmVE, and (**C**) NP+NC marker genes.

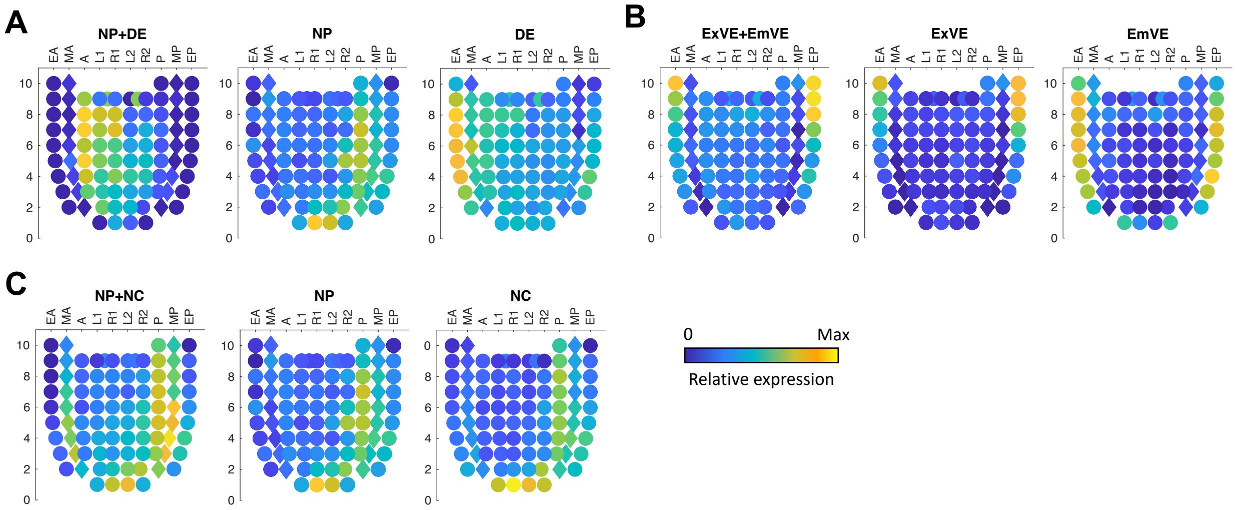

Figure S11. Average expression levels of contact specific marker genes (Supplementary Table S2) on Geo-seq data. (**A**) NP+DE, (**B**) ExVE+EmVE, and (**C**) NP+NC.

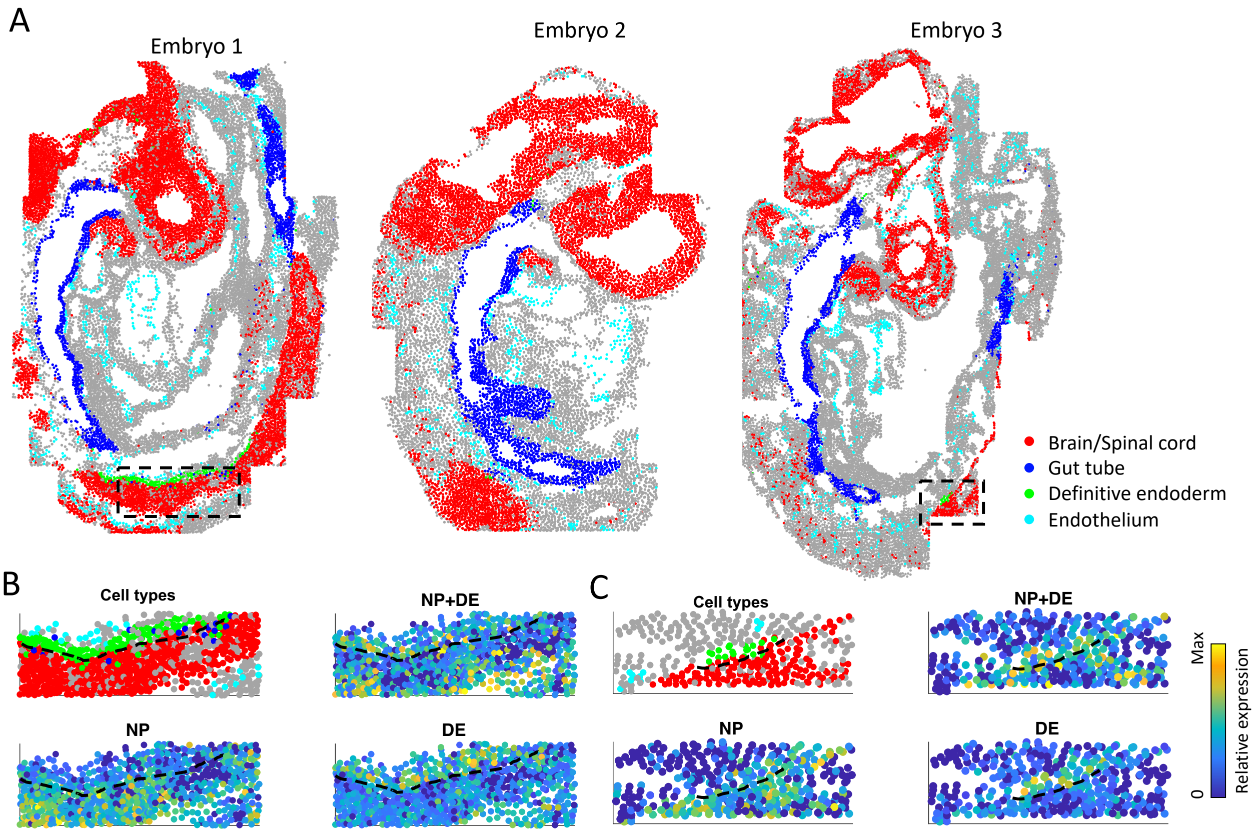

Figure S12. The anatomical position of cell types and average expression profile of contact-specific genes in mouse embryo E8.5-E8.75 on seqFISH data. (**A**) Anatomical position of cell types (Brain/Spinal cord (or NP), Gut tube, Definitive endoderm, and Endothelium) in the mouse embryo (**B**-**C**) Average expression profile of contact-specific and cell type-specific genes (NP+DE, NP, and DE). The boxed region in A is shown at higher magnification in each panel of B and C, with the dotted line showing the interface between the two cell types and the expression of the contact specific genes is apparent along this border.

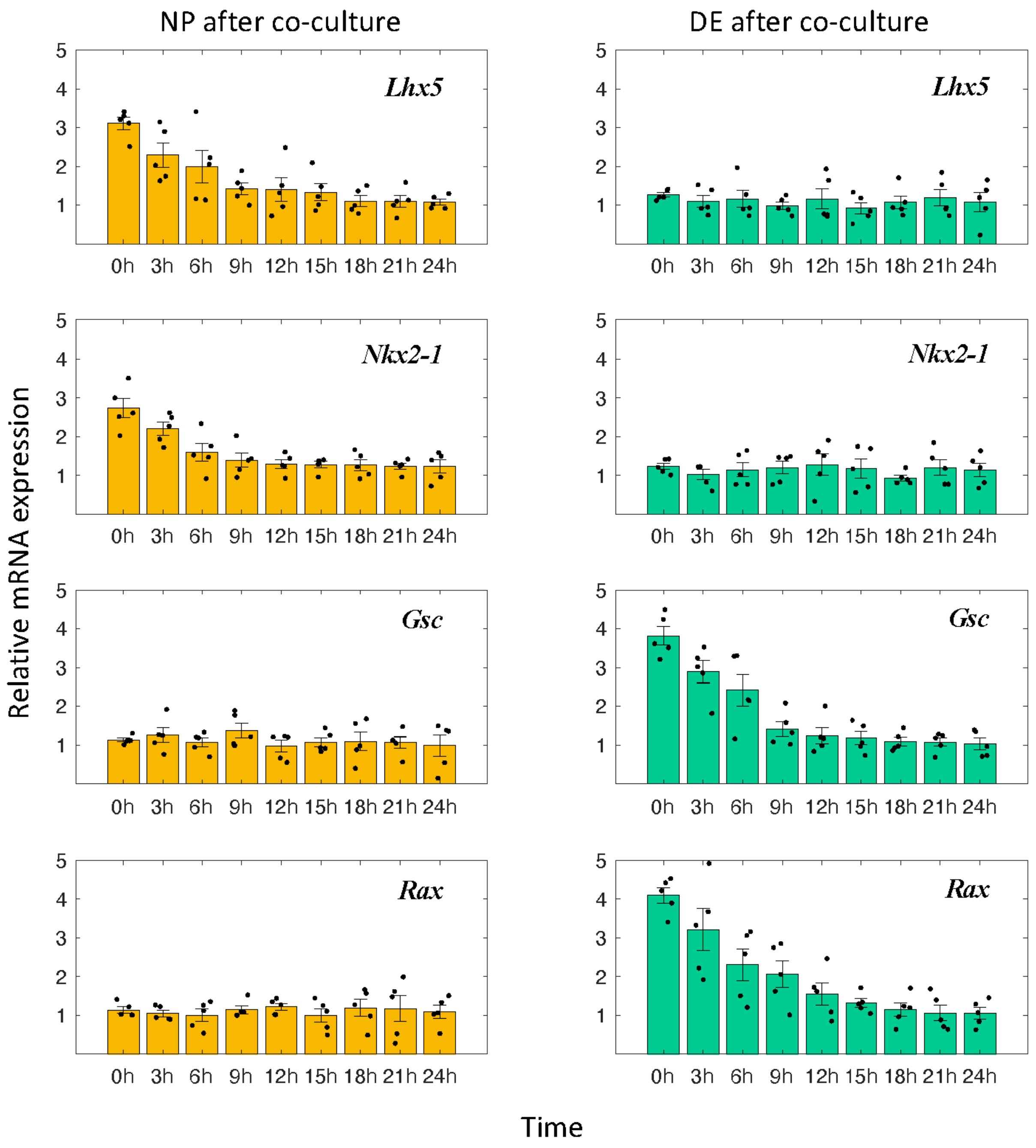

Figure S13. Gene expression changes of the contact specific genes after separation. Expression levels for *Lhx5*, *Nkx2-1*, *Gsc* and *Rax* were measured for the mixed population for NP and DE cells and after separation to each cell type. Expression levels were measured with a 3h interval for 24 hours (*n*=5).

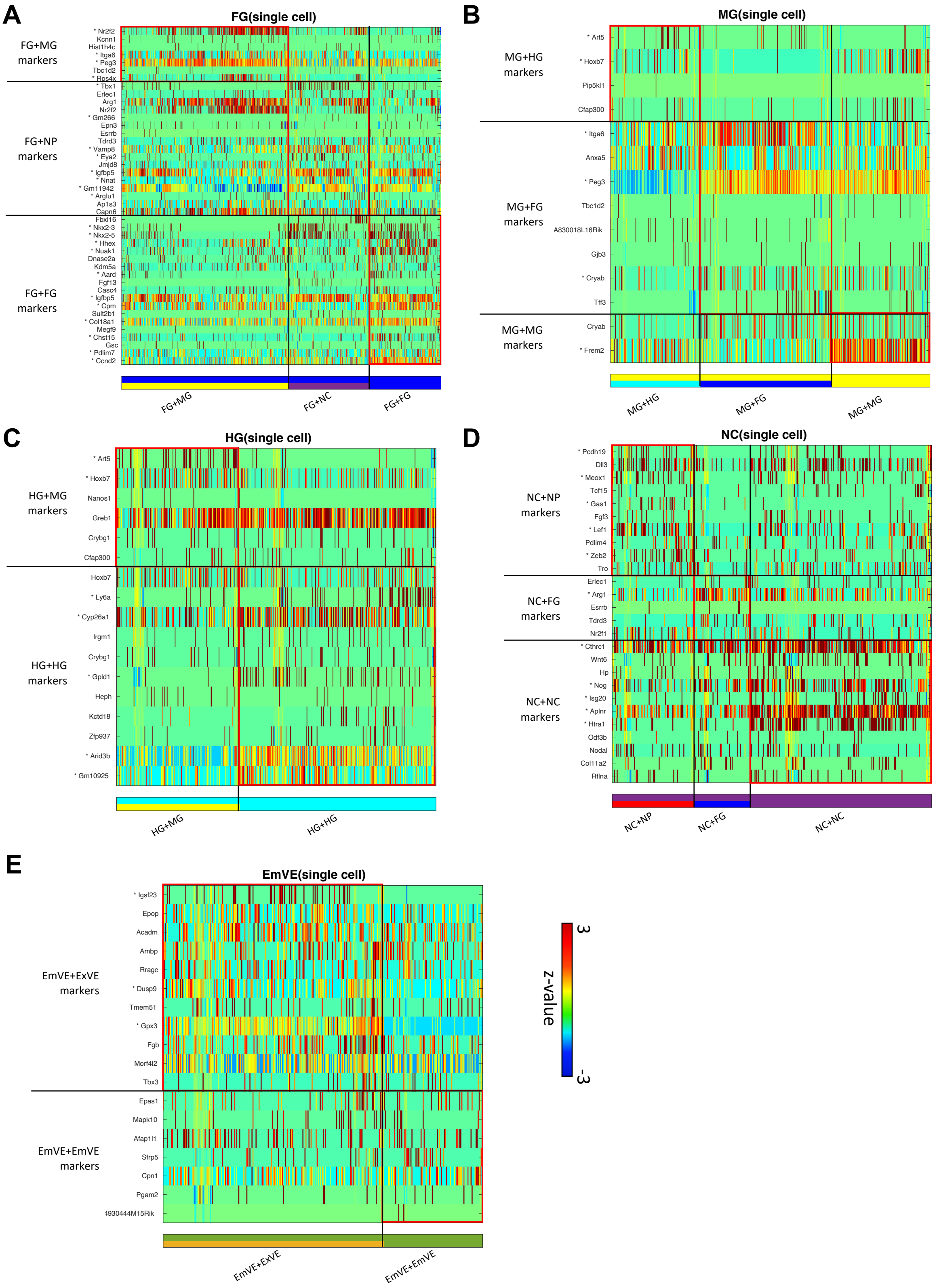

Figure S14. Prediction of the neighboring cell type and the contact-specific expression. (**A**) FG cells predicted to neighbor with MG, NC, and FG, (**B**) MG predicted to neighbor with HG, FG, and MG, (**C**) HG predicted to neighbor with MG, and HG, (**D**) NC predicted to neighbor with NP, FG, and NC, (**E**) EmVE predicted to neighbor with ExVE and EmVE. *: Wilcoxon rank sum test p-value<0.01

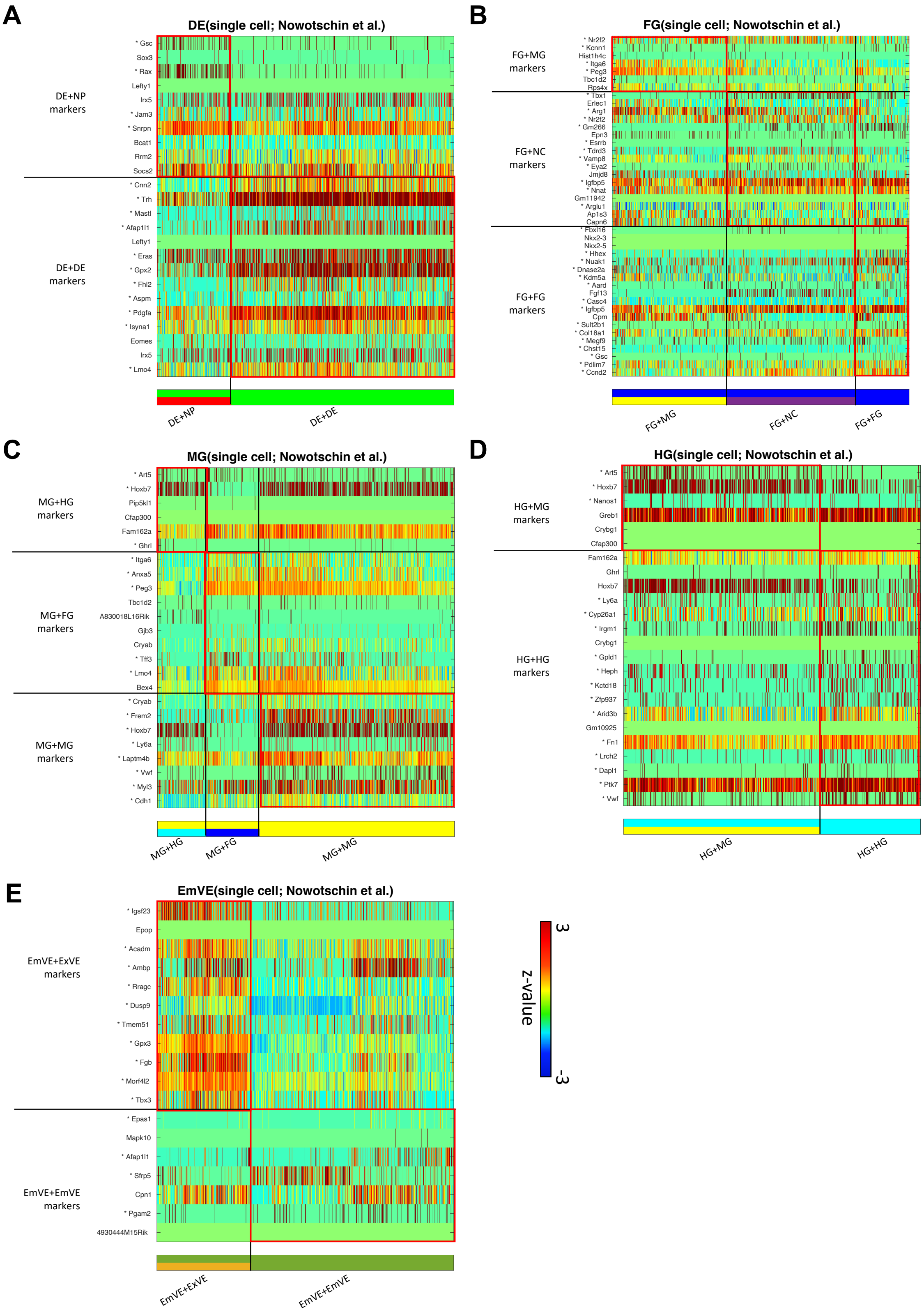

Figure S15. Prediction of the neighboring cell type using an independent publicly available scRNAseq dataset of mouse endoderm during E3.5-E8.75 (Nowotschin *et al.*). (**A**) DE, (**B**) FG, (**C**) MG, (**D**) HG, (**E**) EmVE. *: Wilcoxon rank sum test p-value<0.01.

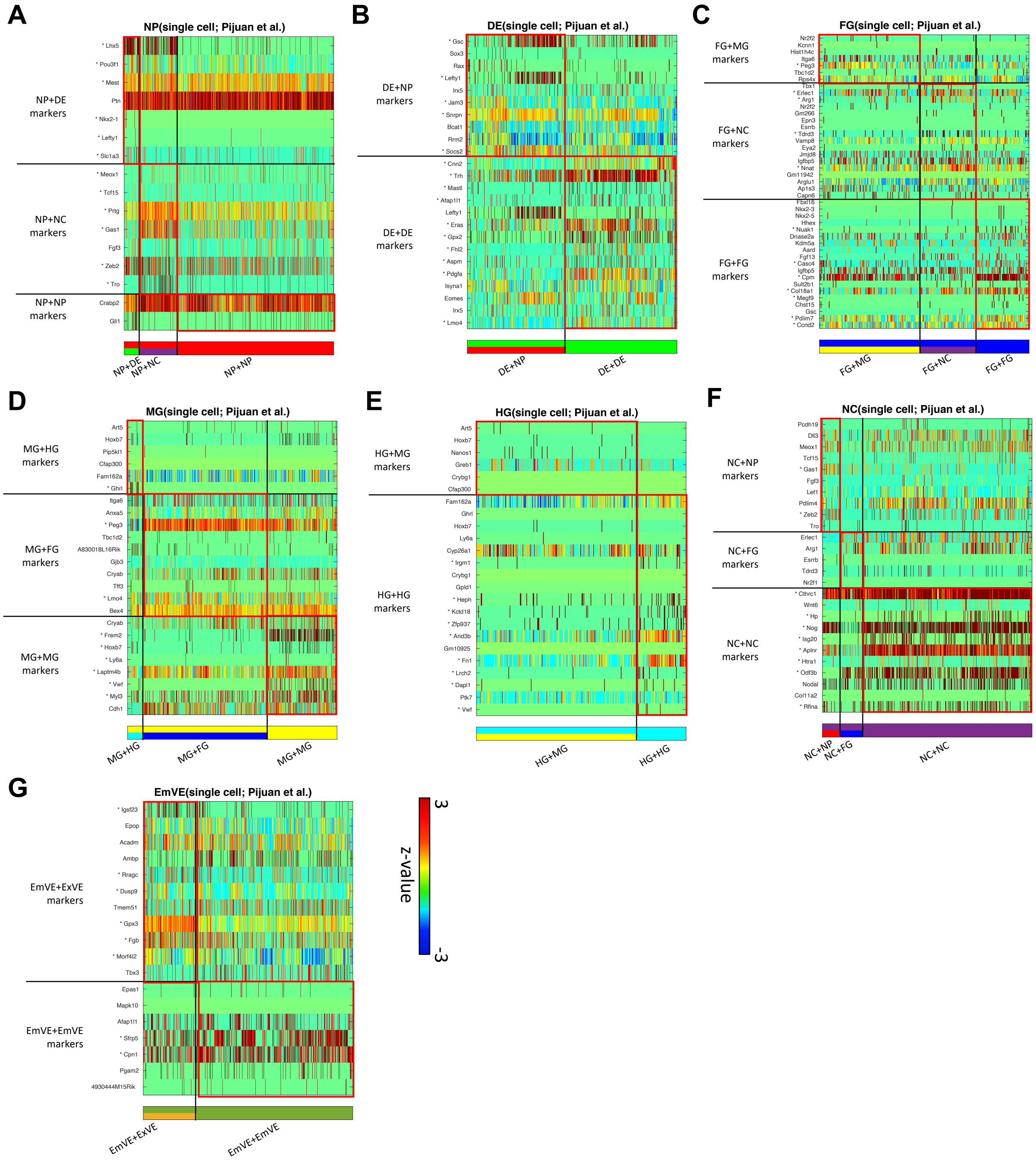

Figure S16. Prediction of the neighboring cell type using an independent publicly available scRNAseq dataset of mouse endoderm during E3.5-E8.75 (Pijuan *et al.*). (**A**) NP, (**B**) DE, (**C**) FG, (**D**) MG, (**E**) HG, (**F**) NC, (**G**) EmVE. *: Wilcoxon rank sum test p-value<0.01.

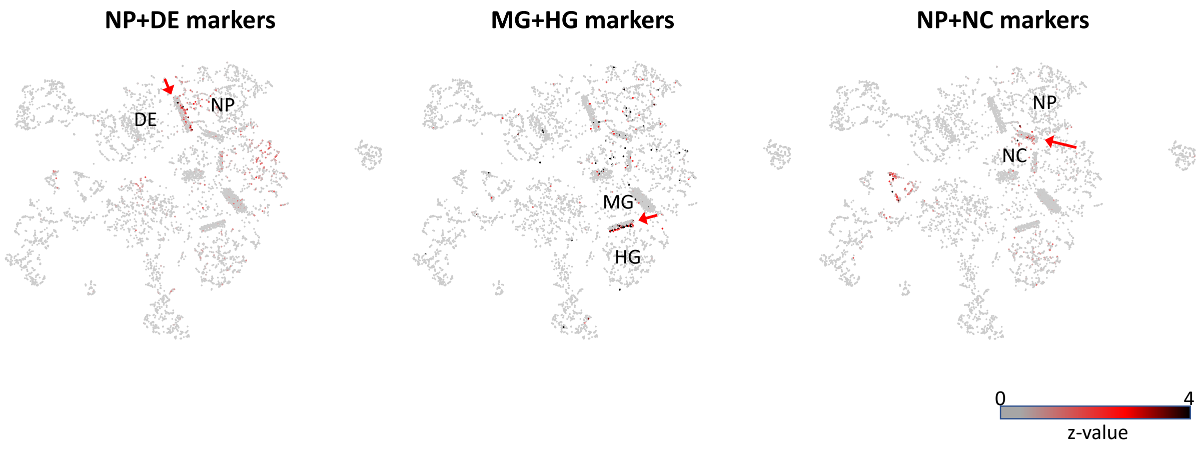

Figure S17. Contact specific marker gene expression on spatial-tSNE. A spatial tSNE plot recapitulates the spatial distribution of cells. The arrows indicate the cells predicted to locate in the border of the corresponding two cell types.

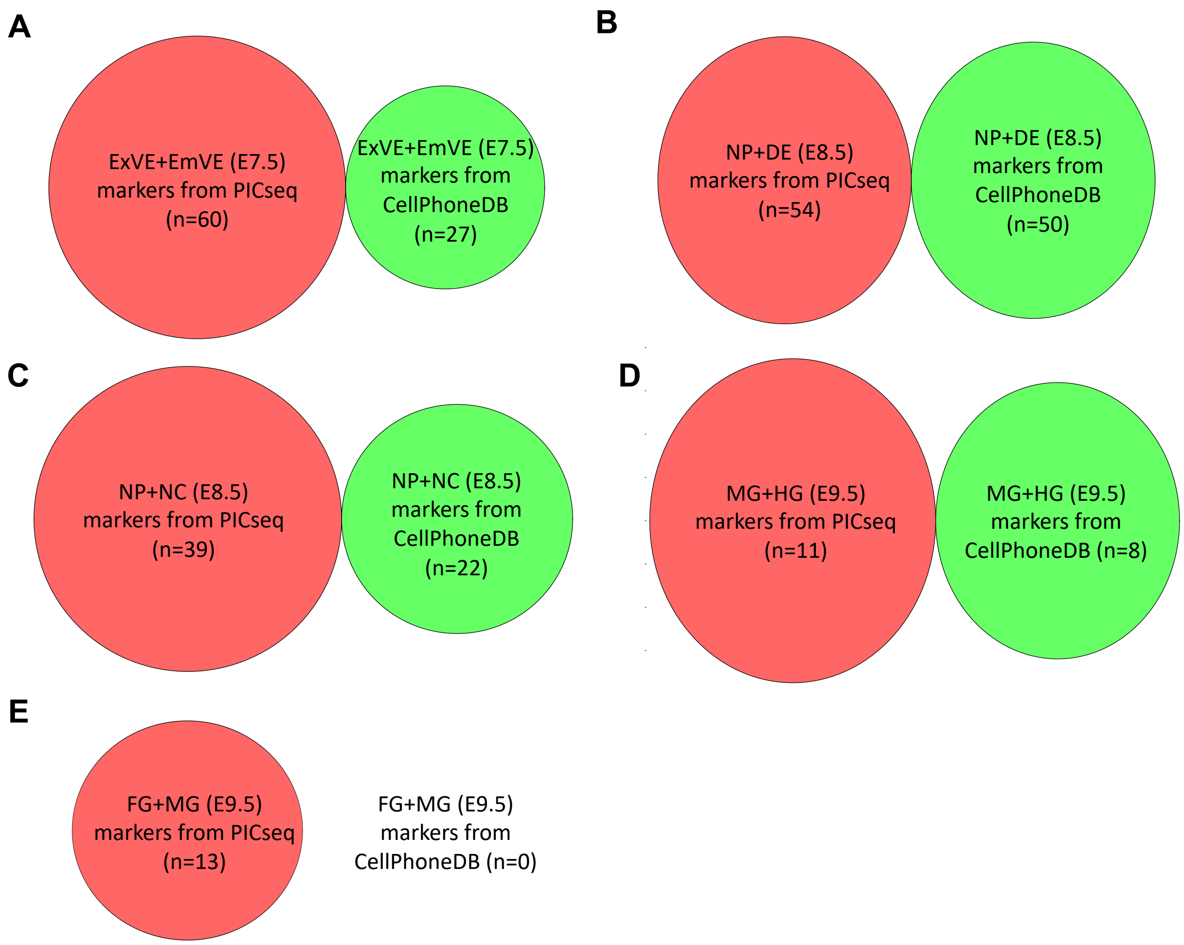

Figure S18. Discrepancy between contact-specific genes identified by PIC-seq and gene sets identified a ligand-receptor analysis CellphoneDB. (**A**) ExVE+EmVE, (**B**) NP+DE, (**C**) NP+NC, (**D**) MG+HG, (**E**) FG+MG.

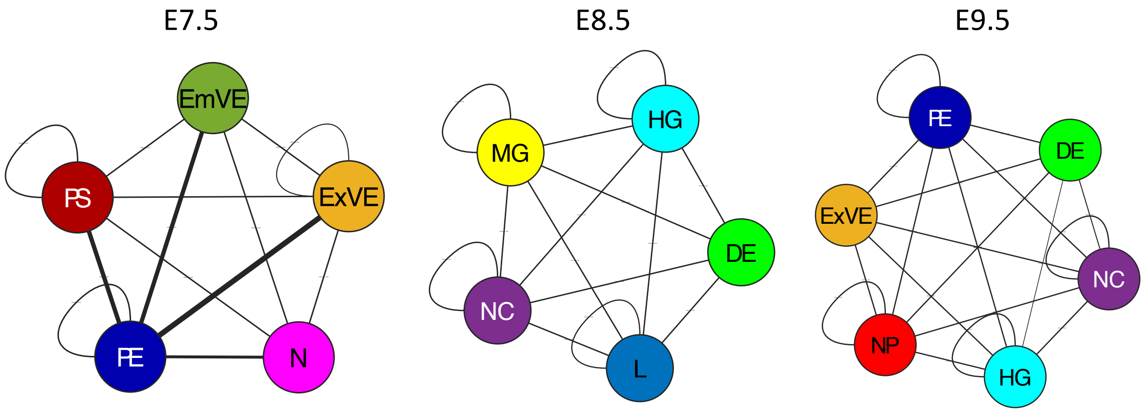

Figure S19. The frequently communicating cell type predicted by CellphoneDB against scRNAseq at E7.5, E8.5 and E9.5. The width of the line represents relative frequencies. The prediction using the ligand-receptor pairs cannot tell the specific cell types interacting with each other.
